# Isolation and Characterization of phiLLS, a Novel Phage with Potential Biocontrol Agent against Multidrug-Resistant *Escherichia coli*

**DOI:** 10.3389/fmicb.2017.01355

**Published:** 2017-07-21

**Authors:** Luis Amarillas, Lucia Rubí-Rangel, Cristobal Chaidez, Arturo González-Robles, Luis Lightbourn-Rojas, Josefina León-Félix

**Affiliations:** ^1^Laboratorio de Biología Molecular y Genómica Funcional, Centro de Investigación en Alimentación y Desarrollo Culiacán, Mexico; ^2^Laboratorio de Genética, Instituto de Investigación Lightbourn, Cd. Jiménez Chihuahua, Mexico; ^3^Laboratorio Nacional para la Investigación en Inocuidad Alimentaria, Centro de Investigación en Alimentación y Desarrollo Culiacán, Mexico; ^4^Departamento de Infectómica y Patogénesis Molecular, Centro de Investigación y de Estudios Avanzados, Instituto Politécnico Nacional Ciudad de México, Mexico

**Keywords:** bacteriophage phiLLS, genome sequence, *in silico*, biotechnological applications

## Abstract

Foodborne diseases are a serious and growing problem, and the incidence and prevalence of antimicrobial resistance among foodborne pathogens is reported to have increased. The emergence of antibiotic-resistant bacterial strains demands novel strategies to counteract this epidemic. In this regard, lytic bacteriophages have reemerged as an alternative for the control of pathogenic bacteria. However, the effective use of phages relies on appropriate biological and genomic characterization. In this study, we present the isolation and characterization of a novel bacteriophage named phiLLS, which has shown strong lytic activity against generic and multidrug-resistant *Escherichia coli* strains. Transmission electron microscopy of phiLLS morphology revealed that it belongs to the *Siphoviridae* family. Furthermore, this phage exhibited a relatively large burst size of 176 plaque-forming units per infected cell. Phage phiLLS significantly reduced the growth of *E. coli* under laboratory conditions. Analyses of restriction profiles showed the presence of submolar fragments, confirming that phiLLS is a *pac*-type phage. Phylogenetic analysis based on the amino acid sequence of large terminase subunits confirmed that this phage uses a headful packaging strategy to package their genome. Genomic sequencing and bioinformatic analysis showed that phiLLS is a novel bacteriophage that is most closely related to T5-like phages. *In silico* analysis indicated that the phiLLS genome consists of 107,263 bp (39.0 % GC content) encoding 160 putative ORFs, 16 tRNAs, several potential promoters and transcriptional terminators. Genome analysis suggests that the phage phiLLS is strictly lytic without carrying genes associated with virulence factors and/or potential immunoreactive allergen proteins. The bacteriophage isolated in this study has shown promising results in the biocontrol of bacterial growth under *in vitro* conditions, suggesting that it may prove useful as an alternative agent for the control of foodborne pathogens. However, further oral toxicity testing is needed to ensure the safety of phage use.

## Introduction

Foodborne diseases are an important cause of morbidity and mortality worldwide, and their incidence has increased globally (Torgerson et al., 2014). The incidence of some foodborne pathogens continues to increase considerably in many countries and are a serious public health problem. Moreover, the risk of illness associated with these foodborne pathogens is exacerbated by the globalization of food marketing and distribution. Foodborne illness outbreaks have a significant impact on human health and are of great economic significance. A recent report from the Food and Drug Administration (FDA) estimates that each year, the economic costs of foodborne illnesses amount to $152 billion (FDA, 2012). This cost is significantly greater than previous official estimates and demonstrates the serious problems to social and economic systems that foodborne illnesses cause.

*Escherichia coli* is among the most important and widespread foodborne pathogens, and has been a significant public health concern globally (Ahmed and Shimamoto, 2014). In recent years, there has been concern that some strains of *E. coli*, which are often multidrug resistant, have caused multiple foodborne disease outbreaks worldwide related to the consumption of contaminated food (Kemper, 2011; Yamasaki et al., 2015). Furthermore, the emergence of antibiotic-resistant strains cause treatments to fail, making the control of these bacteria a challenge.

Recently, multidrug-resistant *E. coli* strains have been isolated from animal feces on rural farms in Northwestern Mexico (Amézquita-López et al., 2016). Various researchers have argued that the sources of fecal pollution in food are paramount in assessing the potential health risks due to potential exposure to pathogens that are highly virulent to humans, and it becomes necessary remedial action (Scott et al., 2003).

The worldwide emergence of antibiotic-resistant bacterial strains creates the need for implementing means to control these threats. The viral–lytic organisms termed bacteriophages (phages) have reemerged as a promising alternative for the control of pathogenic bacteria (Hagens and Loessner, 2010; Mahony et al., 2011). Furthermore, phages are a resource for several biotechnological applications, including vehicles for vaccines, antimicrobial enzymes and phagetyping, and screening libraries of proteins (Monk et al., 2010; Haq et al., 2012). However, the use of bacteriophages as antimicrobial agents requires a clear understanding of phage biology because it allows an estimation of their potential as an alternative effective method for the control of pathogenic bacteria (Sillankorva et al., 2010).

The T-even type of bacteriophages are known by a strictly lytic (virulent) life style, degradation of the host chromosome, and broad host ranges against pathogenic bacteria (Onodera, 2010). Therefore, these phages may be a candidate as an effective biocontrol agent. However, the morphology of bacteriophages alone is insufficient to determine whether or not a phage would be a good candidate for biocontrol purposes. Phages need to fulfill specific characteristics to be used as biocontrol agents. One of the most important requirements related to the use of phages as biocontrol agents to reduce foodborne pathogens is their host range. A suitable phage candidate for effective biocontrol should have a sufficiently broad host range against a wide variety of strains, which is known as a polyvalent bacteriophage (i.e., a bacteriophage capable of productively infecting different bacterial targets) (Parra and Robeson, 2016). Therefore, polyvalent phages may be suitable candidates for the control of bacterial pathogens.

Additionally, although not strongly correlated, the virion morphology characteristic is another factor that may also be an important criterion for selecting phages for biocontrol applications. Usually, *Myoviridae* phages usually exhibit a broader host range than *Siphoviridae* and *Podoviridae* (Chibani-Chennoufi et al., 2004). However, the classification of bacteriophages has been the subject of discussion and various criteria for the classification of coliphages have been proposed. Usually, classification of phages relied on morphology and type of nucleic acids, but genome-based classification was proposed recently (Chen and Schneider, 2005). Therefore, phage genome analysis has been seen as a powerful and promising alternative for contributed to fill the research gap in the area of the taxonomy of coliphages for the implementation of criterion for selecting effective phage for bacterial control (Hagens and Loessner, 2010).

Phages may encode virulence factor genes. Therefore, the complete genome needs to be sequenced to determine whether bacteriophages are suitable to control pathogenic bacteria and whether it is useful to expand our understanding of phage characteristics (Clark and March, 2006).

The aim of this study was to isolate and characterize a polyvalent phage with a wide spectrum of activity as a potential biocontrol agent of multidrug-resistant strains of *E. coli*. These data can provide valuable information to assess the potential of phages as biocontrol agents against pathogenic bacteria. Detailed data on their morphology, determination of their host range, kinetics of phage replication properties, their bacteriolytic activity and their complete genome sequence are described for this bacteriophage. The elucidation of these characteristics may provide valuable information, including the determination of whether the phage has desirable characteristics for potential biotechnological applications.

## Materials and methods

### Bacterial strains and culture conditions

The bacterial strains used in this study are listed in Table 1. *Escherichia coli* strains were isolated previously from farm animal stool samples (Amézquita-López et al., 2014). All *E. coli* strains were provided by the Food Safety National Research Laboratory (LANIIA) at the Research Center in Food and Development (CIAD). The bacteria were grown in tripticase soy broth (TSB) medium (Bioxon, Mexico) at 37°C under aerobic conditions.

**Table 1:** Wild-type bacterial strains used for the host range spectrum of the bacteriophage phiLLS.

***Escherichia coli* strain**	**Serotype**	**Bacterial lysis**
RM8745	O73:H4	−
RM8746	O73:H4	−
RM8747	O15: NT	+
RM8748	O73: NT	+
RM8749	O20:H4	+
RM8750	O20:H4	+
RM8751	O20:H4	+
RM8752	O75:H8	+
RM8755	O111:H8	−
RM8756	O146:H21	−
RM8757	O146:H21	−
RM8758	O146:H21	−
RM8760	O75:H8	+
RM8761	O146:H21	−
RM8762	O146:H8	−
RM8763	O75:H8	+
RM8764	O75:H8	−
RM8765	O75:H8	+
RM8772	O8:H19	−
RM8773	O8:H19	−
RM8774	O8:H19	−
RM8775	O8:H19	−
RM8776	O8:H19	−
RM8778	O75:H8	+
RM8779	O75:H8	+
RM8780	O75:H8	+
RM8916	O111:H8	−
RM8917	O168: NT	+
RM8929	O75:H8	+
RM8930	O75:H8	+
RM8744	O157:H7	+
RM8753	O157:H7	+
RM8754	O157:H7	+
RM8759	O157:H7	+
RM8767	O157:H7	+
RM8768	O157:H7	+
RM8769	O157:H7	+
RM8771	O157:H7	+
RM8781	O157:H7	+
RM8921	O157:H7	+
RM8922	O157:H7	+
RM8927	O157:H7	+
RM8928	O157:H4	+
RM9450	O157:H7	+
RM9451	O157:H7	+
RM9452	O157:H7	+
RM9453	O157:H7	+
RM9454	O157:H7	+
RM9455	O157:H7	+
RM9456	O157:H7	+
RM9457	O157:H7	+
RM9458	O157:H7	−
RM9459	O157:H7	−
RM9460	O157:H7	+
RM9461	O157:H7	+
RM9462	O157:H7	−
RM9463	O157:H7	+

*Phage was assessed for host range by spot testing. (+) indicate positive sensitivity to phage lysis, and (−) indicate negative sensitivity to phage lysis. E. coli strains were isolated previously from farm animal stool samples (Amézquita-López et al., 2014)*.

### Bacteriophage isolation and purification

Pond water and wastewater samples were collected between November and December 2015 in different regions in Sinaloa, Mexico. The samples were assayed for the presence of phages capable of forming plaques on *E. coli* strains. Phages were detected by the plaque assay method previously described by Jamalludeen et al. (2007), with slight modifications. Briefly, water samples were centrifuged at 8,500 × *g* for 15 min and the supernatant filtered through a 0.22-μm pore membrane. Then, 100 μL of the filtered water sample was added to 1 mL of logarithmic phase. *E. coli* O157:H7 CECT 4076 and mixed with 3 mL of pre-warmed TSB top agar (0.4% agar), spread on TSA plates, and incubated overnight at 37°C. The plates were checked for plaques and large, clear and non-turbid plaques by phage were selected and picked from the TSA plates. Subsequently, purified plaques were diluted in nanopure water and stored at 4°C. This procedure was repeated three times to obtain single-plaque isolates.

### Transmission electron microscopy

Electron micrographs of purified phage particles were obtained according to standard method. Suspension phage sample was dropped (approximately 30 μL) onto 400-mesh carbon-coated Formvar covered grids placed in a vacuum evaporator (JEE400, JEOL Ltd. Tokyo, Japan), stained with 2% (wt/vol) phosphotungstic acid (pH 7.2), and air dried. Samples were examined in a transmission electron microscope (JEM-1011, JEOL Ltd. Tokyo, Japan) at an acceleration voltage of 80 kV, and phage particles were examined at 15,000–25,000 times magnification.

### Determination of the host range

The host range of the phage was tested against 57 strains environmental isolated by the spot method (Kutter, 2009). The bacterial strains used in this essay are listed in Table 1. The host specificity of the phage was determined by the spot method. One milliliter of an overnight culture of each tested bacterium (~10^8^ CFU mL^−1^) was added to 3 mL of molten TSB top agar (0.4% agar). The mixture was then overlaid on trypticase soy agar plates. The plates were allowed to dry for 30 min at room temperature and a 10-μL drop of each serially diluted phage lysate was spotted onto the surface of the plates, followed by overnight incubation at 37°C. Subsequently, plates were examined visually for clearance zones and the presence of a lytic zone was considered evidence of bacterial susceptibility to phage-mediated lysis.

### One-step growth curve

The experiment to determine the latent period and phage burst size was carried as described previously (Goodridge et al., 2003), with minor modifications. *Escherichia coli* O157:H7 CECT 4076 strain was grown in 40 mL of TSB at 37°C to an OD_600_ of 0.1 (~10^8^ CFU mL^−1^). Phage was added at a multiplicity of infection (MOI) of 0.001 and allowed to adsorb for 5 min at room temperature. After the phage adsorption period, the mixture was centrifuged at 8,500 × *g* for 1 min to remove any non-absorbed phage. The supernatant was discarded, the pellet was resuspended in 40 mL of fresh TSB and incubated with shaking (200 rpm min^−1^) at 37°C. Samples were obtained at 10 min intervals, and immediately centrifuged at 8,500 × *g* for 1 min and then the supernatant was diluted and plated for phage titration determined by the double-layer agar plate method, expressed as plaque-forming units per milliliter (PFU mL^−1^). The data from the plaque assays were analyzed. The number of PFU mL^−1^ vs. time was plotted using Excel and the latent period and burst size were determined. All experiments were performed at least in triplicate.

### Bacterial challenge test

Phage bacteriolytic activity was determined *in vitro* as previously described by Wang et al. (2016), with some modifications. Briefly, *E. coli* O157:H7 was incubated into TSB medium and grown overnight at 37°C, and a subsequent 1 mL of culture was transferred to 50 mL of fresh TSB and incubated at 37°C with shaking at 200 rpm until the OD_600_ reached 1.0. Thereafter, phage was added at an MOI of 0.1, 1.0, and 100. Bacterial growth was monitored by turbidity measurements every 30-min interval for 4 h using OD_600_ nm. All experiments were performed at least in triplicate. Statistical significance was determined using Student's *t*-test in Microsoft Excel 2010 with a *P*-value threshold of ≤0.05.

### Bacteriophage propagation and DNA extraction

Phage phiLLS was propagated using the double agar overlay technique as described previously by Jamalludeen et al. (2007). Briefly, a culture of *E. coli* O157:H7 was grown at 37°C in tryptic soy broth (BD Bioxon, Mexico) overnight. One milliliter of *E. coli* culture and 100 μL of phage phiLLS stocks were mixed with 3.8 mL of TSB top-agarose (0.4%). The top-agarose was overlaid on a tryptic soy agar plate and placed at rest to solidify. After incubation at 37°C for 18–24 h, 6 mL of Suspension Medium (SM buffer) [Tris-HCl, 50 mM, pH 7.5; MgSO_4_ 7H_2_O, 8 mM; NaCl 100 mM; gelatin 0.002 % (p/v)] were added for eluting the top agar overlaid plates. The eluate was recovered and centrifuged for 15 min at 15,000 × *g* and the supernatant was filtered through 0.45-μm sterile syringe filters (Whatman, UK). The resulting filtrate was centrifuged for 2 h at 40,000 × *g*. After centrifugation, the supernatant was discarded and the pellet was resuspended by pipetting in 10 mL of SM buffer and filtered through 0.22-μm sterile syringe filters of cellulose acetate membrane (GVS, USA). Finally, the phage suspension concentrate was stored in the dark at 4°C. Five milliliters of phage suspension were used for DNA extraction. The phage suspension was incubated with 10 μL of DNase I/RNase A (10 mg/mL^−1^) (Sigma-Aldrich, USA) at 37°C for 30 min, followed by the phage DNA isolation using the SDS-proteinase K protocol as described previously by Sambrook and Russell (2001).

### Determination of the bacteriophage genome ends

The identification of phage packaging strategies, and the type of physical ends of bacteriophage genome can often be deduced based on phylogenetic analysis of amino acid sequences of terminase large subunit of phage compared to other phages with known DNA packaging strategies (Wittmann et al., 2014). Therefore, in this study, the large terminase subunit amino acid sequence was used to reconstruct the phylogenetic tree in order to analyze the phylogenetic relationship among phage phiLLS and other phages.

The predicted amino acid sequences of the large terminase subunits genes of dsDNA coliphages were retrieved from National Center for Biotechnology Information (NCBI) and were used for phylogenetic analysis. The bacteriophages included in this study has been molecularly analyzed independently from investigators throughout the world and contains the well-characterized dsDNA bacteriophages with different types of packaging strategies depend on terminase actions (headful, 5′-extended cos ends, 3′-extended cos ends and direct terminal repeats) experimentally determined. The phage large terminase proteins included are listed below with their respective accession numbers. All sequences were aligned using ClustalW in Geneious with default parameters. Phylogenetic trees were inferred using neighbor-joining algorithm and statistical support for the internal nodes was determined by 1,000 bootstrap replicates in Geneious version R9.

Additionally, the genome ends were determined as described by Casjens and Gilcrease (2009). To detect the presence of cohesive (*cos*) genome ends, approximately 1 μg phage DNA was digested with specific restriction enzymes (*Eco*RI, *Eco*RV, *Bam*HI, and *Hind*III) according to the manufacturer's guidelines (Promega, USA). After 1 h of incubation at 37°C, the reaction mixture was divided into two equal proportions aliquots. Both aliquots were then incubated at 75°C for 15 min. Subsequently, one aliquot was rapidly cooled on ice. The second aliquot was allowed to cool slowly to room temperature, because under these conditions the potential complementary cohesive ends can be annealed. To analyze DNA fragments, the molecules were separated by agarose gel electrophoresis (1% w/v) in TAE electrophoresis. The gel was stained with ethidium bromide and visualized by UV illumination. *Hind*III-digested Lambda DNA marker (Promega, USA) was used as a control because it contains cohesive (cos) ends and used to estimate DNA fragment sizes.

### Detection of genes encoding *Stx* by PCR

Detection of the genes encoding Shiga toxin 1 (*stx*1) and Shiga toxin 2 (*stx*2) in nucleotide sequence of phage was performed by multiplex PCR as previously described by Paton and Paton (2002). Amplification was performed with a CFX96 PCR system (Bio-Rad laboratories, Inc., USA) using a GoTaq® PCR Core System I (Promega, USA) in a total volume of 25 μL containing each dNTP at 100 μM, 1.5 mM MgCl_2_, 10 pmol of each primer, 5 × GoTaqBuffer, and 1 U GoTaq polymerase (Promega, USA) according to the manufacturer's instructions. One microliter of purified phage DNA was used as templates in PCR assays. As an internal positive control of PCR, *E. coli* O157:H7 CECT 4076 DNA was included in the assays. Two microliters of each PCR product was analyzed by agarose (0.8%) gel electrophoresis, and bands were viewed by ethidium bromide staining. The primers set used in the PCR assays were commercially custom-synthesized by Sigma-Aldrich (Toluca, Mexico).

### Genome sequencing and annotation

The phage genome was sequenced using a TruSeq protocol on an Illumina HiSeq platform, with pair-end read sizes of 100 bp. The raw reads were quality checked through FastQC and trimmed with FASTQ Quality Trimmer (minimum Q30 score) available on the public Galaxy server (https://usegalaxy.org/). Quality-controlled trimmed reads were *de novo* assembled to a single contig with 120-fold coverage using Geneious 9.0.5 (Kearse et al., 2012). Potential ORFs were predicted using GeneMark (http://exon.gatech.edu/) and ORF Finder (http://www.ncbi.nlm.nih.gov/gorf/gorf.html) using the bacterial, archaeal and plant plastid code (transl_table = 11). Functional annotation was screened using BLASTP and Psi-BLAST algorithms against the non-redundant protein database at NCBI. The genome of phage was scanned for tRNAs using tRNAscan-SE (Lowe and Chan, 2016) and Aragorn (Laslett, 2004). Additionally, the deduced amino acid sequences of all the ORFs were analyzed using the NCBI Conserved Domain Database, HMMER, Prosite, SMART and Motif Search to detect conserved motifs among the proteins. Potential promoters were predicted using the Neural Network Promoter Prediction tool of the Berkeley Drosophila Genome Project (http://www.fruitfly.org/seq_tools/promoter.html), considering only sites located in intergenic regions (Reese, 2001) and Rho-independent transcription terminators were predicted using FindTerm program (http://linux1.softberry.com/berry.phtml?topic=findterm&group=programs&subgroup=gfindb; energy threshold value: −11) and ARNold server (http://rna.igmors.u-psud.fr/toolbox/arnold/), respectively (Naville et al., 2011). The codon usage of the phage genome was determined with the Geneious software and was compared with the codon usage of the *E. coli* genomes available in the NCBI database. Additionally, comparative genomic analysis of phage isolated with homologous phages was conducted with progressive Mauve alignment to determine conserved sequence segments of the phage genomes. Moreover, cumulative GC skew analysis was performed with GenSkew-genomic nucleotide skew application (http://genskew.csb.univie.ac.at/). The completed genome sequence of phage phiLLS has been deposited in the GenBank database under accession number KY677846.1. The graphical representation was made with Excel.

## Results and discussion

### Phage isolation and morphology

Several water samples, which included ponds, creeks, streams, and canal ways, were tested for the presence of bacteriophages against *E. coli*. A phage, designated phiLLS, was isolated from a water sample collected from a pond (located in the southwest region of Culiacan, Mexico at coordinate [24°35′30.2″N 107°26′26.2″W]) using the double-layer agar assay technique. Our results coincide with Reyes and Jiang (2010) who explained that the presence and replication of lytic phages in environmental water, suggesting that coliphage replication in this type of environment may become significant because the *E. coli* strains isolated from environmental water are sensitive to somatic coliphages. This finding is supported by the fact that several investigations have reported the presence of lytic phages in environmental water that are specific for *E. coli* (Begum et al., 2009).

phiLLS formed clear plaques, with sizes ranging from 1.5 to 2.0 mm in diameter, and well-defined boundaries against the *E. coli* bacterial host strain. Plaques were obtained after incubation of plates at 37°C overnight. According to Abedon and Yin (2009), the morphology and plaque size may vary in size depending on growth conditions, but typical virulent phages produce clear plaques, whereas phages with the ability to lysogenize form turbid plaques, supporting the idea that phage phiLLS may be preliminarily considered a virulent phage.

**Figure 1:**
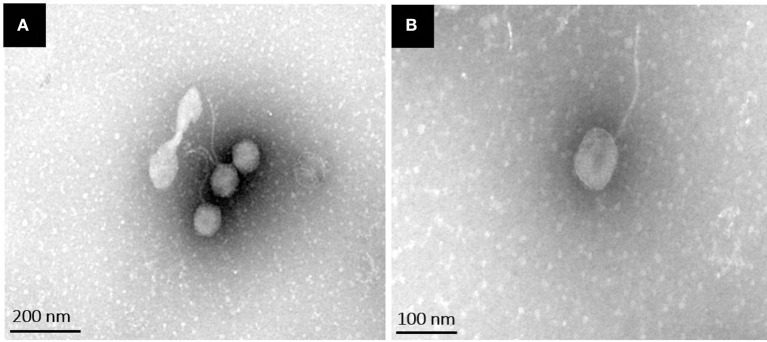
Transmission electron microscopy images of phiLLS negatively stained using 2% uranyl acetate. Negatively stained electron micrographs of phiLLS virions showing the typical morphology of phages within the family *Siphoviridae*. **(A)** Broad view of the phage particles. **(B)** High magnification of a single phage particle.

Transmission electron microscopy analysis revealed that phiLLS had an isometric and icosahedral head with an estimated diameter of 56 ± 2 nm. The phage presented a non-contractile, long flexible and extremely thin tail, measuring 135 ± 5 nm in length and 15 ± 1 nm in width. The presence of a neck, a base plate, spikes, or fiber, is not seen in the mature phage. To date, bacteriophages are classified based on differences in the morphology of their virion characteristics. According to their morphological characteristics and based on guidelines of the International Committee on Taxonomy of Viruses (Fauquet et al., 2005), phage phiLLS belongs to the family *Siphoviridae* in the order *Caudovirales* (Figure 1). Over 95 % of the phages reported in the scientific literature belong to the *Caudovirales* (tailed phages) (Bebeacua et al., 2013). Almost (60%) all the bacteriophages described are phages with long and flexible tails assigned to the family *Siphoviridae* (Ackermann, 2006). In accordance, the phage phiLLS belongs to this taxonomic classification.

### Detection of the *stx* genes

Shiga toxin (Stx) is one of the most potent bacterial toxins, and genes encoding these toxins are located on different bacteriophages, which are integrated into the bacterial chromosome (Mauro and Koudelka, 2011). Therefore, to satisfy the selection criteria for the use of phages as a biocontrol agent, the phage must be assessed for the absence of the genes associated with virulence factors. A preliminary analysis of the phage genome was conducted to amplify genes encoding the different Stx using PCR assay. Genes for *stx* were not detected in the phiLLS genome sequence. The lack of *stx* genes in the phage suggests that it may be safe for use in biocontrol. However, the possible presence of other virulence factors genes that may contribute to virulence should be evaluated, as coliphages may carry genes coding for diverse virulence factors such as intimin, enterohemolysin or human serum amyloid A (Kelly et al., 2009). Therefore, it is essential to analyze the complete sequence of phiLLS to ensure that the genome of bacteriophage did not contain any detrimental genes encoding, for example, genes associated with the development of antibiotic resistance, lysogenic proteins, toxins or other virulence factors, providing a comprehensive assessment of phage safety based on their complete genome sequences.

### Broad host range

The ability of new isolated phage to lyse pathogenic *E. coli* strains was assayed by the spot test. These strains belong to the Shiga toxin-producing *E. coli* (STEC) pathotype, including six different somatic O antigens (O157, O15, O73, O75, O20, and 168) and three different flagellin H antigens (H8, H4, and H7) (Table 1). These strains are resistant to multiple antibiotics (amoxicillin, clavulanic acid, amikacin, cephalothin, chloramphenicol, imipenem, kanamycin, and tetracycline; Amézquita-López et al., 2016). Phage suspensions produced an inhibition halo on 39 of the 57 (68.42%) strains tested, which is considerably higher than the infection rate of approximately 40% observed in other coliphages also isolated from water samples and recently reported (Ghasemian et al., 2017; Hamdi et al., 2017).

Based on the host range studies, the newly isolated phage phiLLS possesses a broad lytic spectrum. The broad host range infectivity against a diverse collection of *E. coli* isolates, showing that phiLLS is a polyvalent phage on different strains of importance for human and animal health. Phages are usually highly specific; most can infect only a single species of bacteria. However, some polyvalent bacteriophages have been reported (Hamdi et al., 2017). These polyvalent phages can infect various host species, making them the most promising candidates for biocontrol development. Therefore, our data suggest that the phage phiLLS may be a promising potential candidate as a biocontrol agent against *E. coli*.

### One-step growth curve

**Figure 2:**
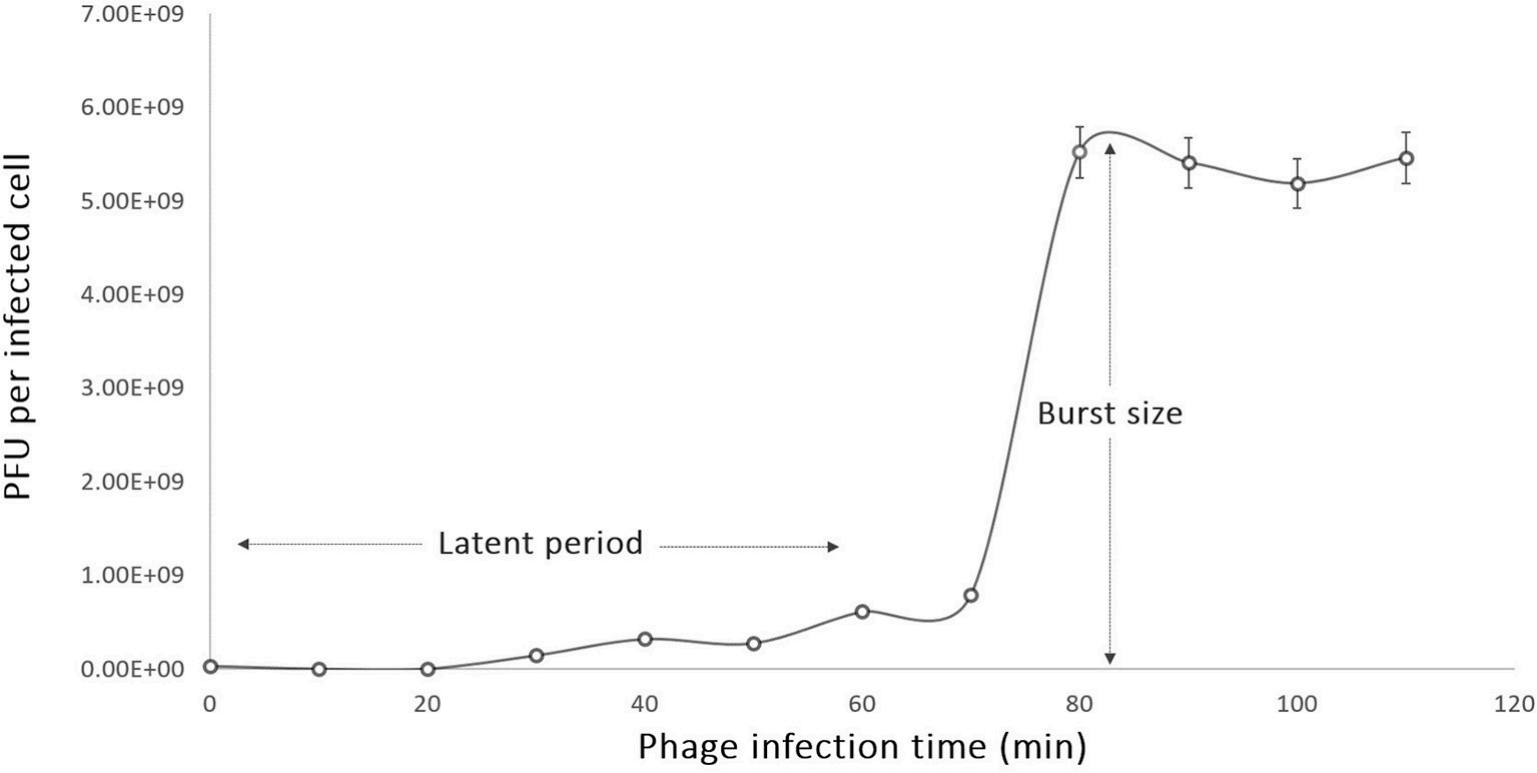
One-step growth curve of phage phiLLS. Shown are the pfu per infected cell in the cultures at different time points. Each data point represent mean from three independent experiments, and the error bars indicate standard deviations. **(A)** The latent period is 15 min and **(B)** burst size was estimated to be 176 PFU per one infected cell.

The one-step growth studies were conducted to investigate the different phases of the phage infection process such as the latent period and the burst size of phage phiLLS. According to the one-step growth experiment, the latent period of phiLLS propagated on *E. coli* was approximately 70 min, the rise period was 12 min, and the average burst size was estimated to be 176 plaque-forming units (PFU) per infected cell (Figure 2). The large-scale biocontrol of bacterial pathogens requires phages of high lytic activity against high numbers of bacterial target cells, a feature that is correlated with the large burst size. Large burst size is considered one of the major characteristics of an effective bacteriophage as antimicrobial agent because burst size is closely related to phage propagation (Gallet et al., 2011). A phage with a large burst size may have a selective advantage as an antibacterial agent since phages with a large burst size can increase the initial dose of phages several 100-fold in short periods of time (Choi et al., 2010; Nilsson, 2014). Therefore, the large burst size of phiLLS can be a definite advantage for its application as the biocontrol agent against bacterial pathogens.

### Bacterial challenge test

**Figure 3:**
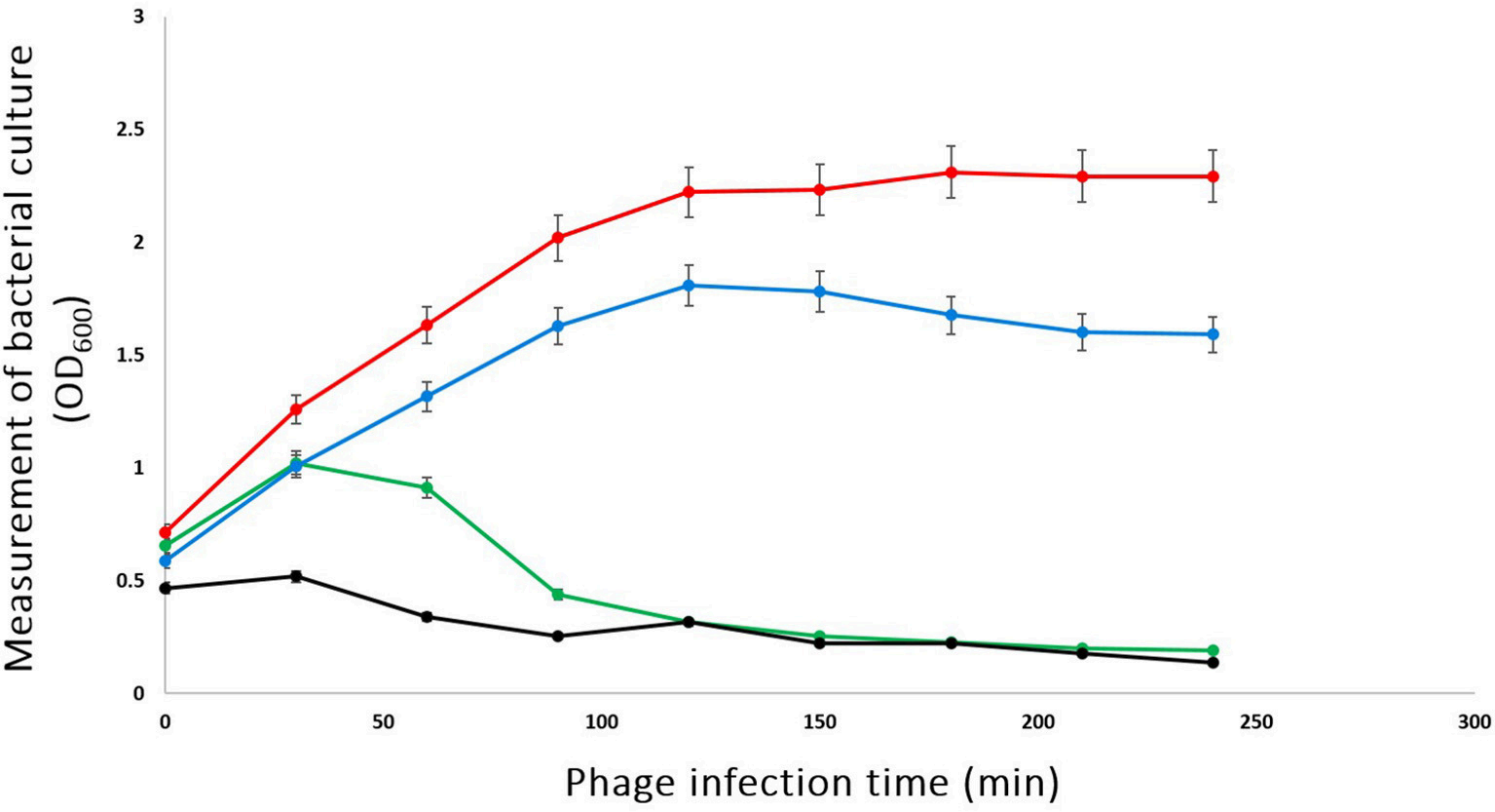
Bacterial challenge test of phage phiLLS with *E. coli* O157:H7 CECT 4076. *E. coli* log-phase culture was infected with phage phiLLS at 100 (Line black), 1.0 (Line green), and 0.1 (Line blue), when the OD at 600 nm was 1.0. The growth curve of bacterial was used as a control (Line red). The graphs show viable-cell counts of samples collected every 30 min. The error bars indicate standard deviations from the results of triplicate experiments.

To investigate the ability of phage phiLLS to lyse *E. coli* O157:H7 in *in vitro* culture conditions, challenge tests were performed that included the addition of phage at an MOI of 0.1, 1.0, and 100 to mid-exponential-phase cells around OD_600_ reaching 1.0 (Figure 3). Significant decreases in the viability of bacterial strains were observed, mainly in cells infected with an MOI of 1.0 and 100, but the reduction of bacterial cells was not so at an MOI of 0.1. These data showed that at lower phage concentrations, bacterial concentration started to increase, possibly due to many cells are not infected and continued to divide. Four hours after phage addition at an MOI of 1.0 and 100, a 3-log-unit (1,000-fold) reduction in the number of viable cells was observed when compared with the control where no phage had been added. Expressed differently, for the addition of the phage at an MOI of 1.0 and 100, the viable bacterial counts of *E. coli* decreased by 94 % over the course of the experiment. The *in vitro* challenge test demonstrated that the phage phiLLS could be used to inactivate strains of pathogenic *E. coli* and has the potential to be used as biocontrol agent. The *in vivo* challenge test will be the next focus on our research. However, it is possible that after time, the bacteria will be able to regrow, due to the emergence of a host population that was able to resist phage lysis or bacterial insensitive mutants (BIMs), as has been reported by several authors (Yordpratum et al., 2010; Yamaki et al., 2014). To solve this problem, phage phiLLS can be mixed with other phages in a phage cocktail. This may be effective to control the appearance of phage-resistance cells, since BIMs emergence can be bypassed by adding others with different infection mechanisms (Yamaki et al., 2014).

In general, the results of host-cell lysis caused by phage phiLLS demonstrated that the bactericidal activity was related to the MOI. In this study, the MOI 100 ratio showed the highest reduction rate of viable bacteria count. Previous studies also found that the higher MOI resulted in lower numbers of viable bacteria. However, it is also important consider that high MOI effects may attenuate bacteriophage proliferation in natural systems, which that as the result of adsorption of large numbers of phage causing destabilization of the outer membrane and subsequent bacterial lysis, preventing phage replication and release “lysis from without” (Brown and Bidle, 2014).

### Bacteriophage genome ends

The ability of bacteriophages to facilitate horizontal gene transfer through transduction is an important consideration for using them for the control of pathogenic bacteria (Meaden and Koskella, 2013). The mechanism by which dsDNA is packaged into the bacteriophage determines how this process may occur (Horgan et al., 2010).

**Figure 4:**
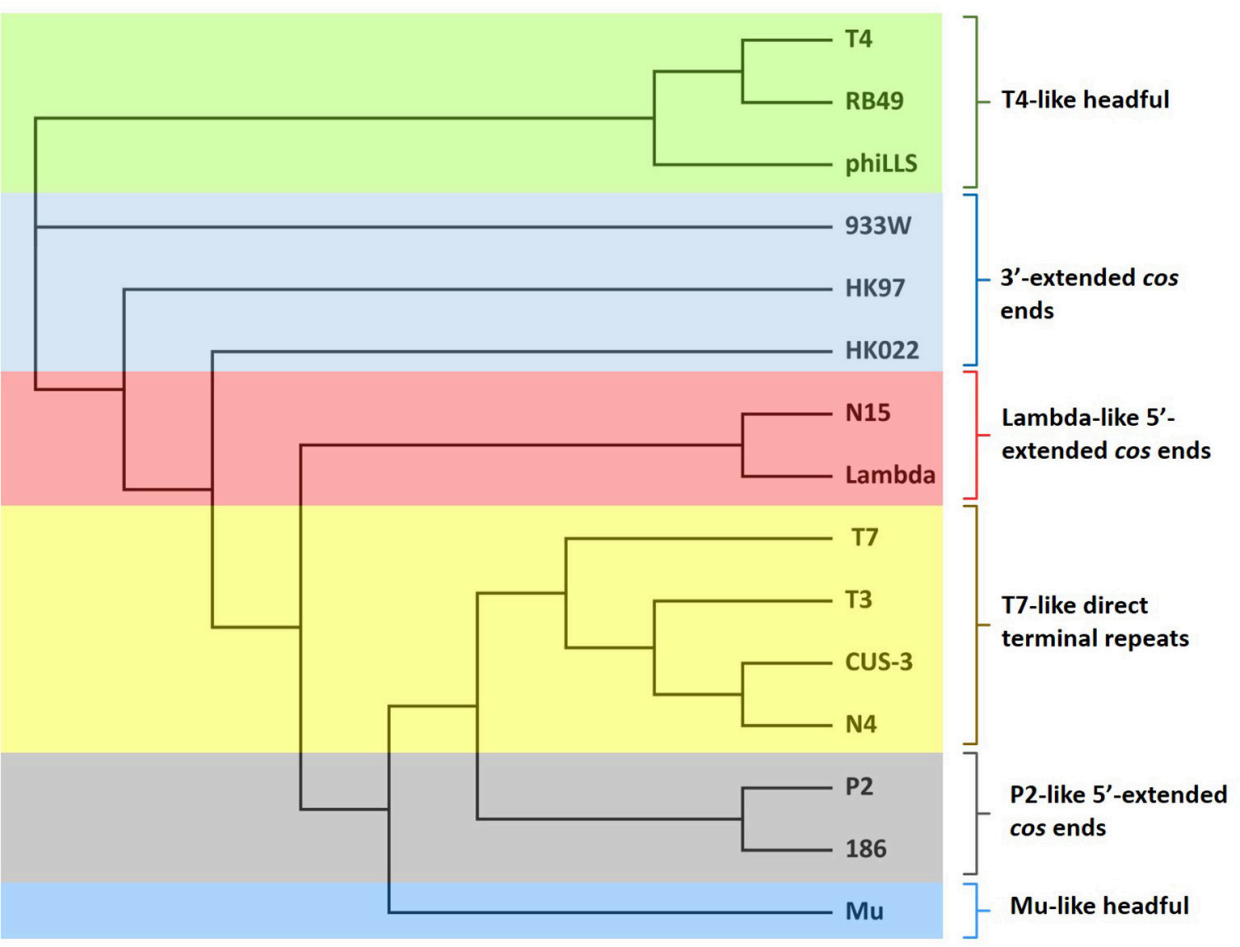
Neighbor-joining phylogenetic tree of terminase large subunit of phiLLS and their comparison to other coliphages with known packaging mechanisms. Bootstrap analysis was performed with 1,000 repetitions. The terminase large subunits were compared using the ClustalW in Geneious program version R9. Colored boxes indicate the phages grouped into similar cluster that share same packaging strategy.

The phylogenetic analysis of the large terminase subunit suggests that phiLLS is a headful packaging phage containing a circularly permuted genome (Figure 4). Based on phylogenetic analysis of the large terminase subunit, this phage was predicted to be *pac*-type phage using a head-full DNA packaging strategy. This result is not surprising, as most phages follow the headful packaging mechanism (Molineux and Panja, 2013).

According to the results obtained, phiLLS was clustered with the terminases of phages RB49 and T4, both with DTR in their chromosome ends, this cluster share high identity indicates strong phylogenetic relationship between theses phages. Based on the close association with the large terminases of phages that have an experimentally confirmed packaging strategy, it is predicted that the genome of the phiLLS has possibly circularly permuted direct terminal repeats. To support this finding, the phage genome was treated with different restriction enzymes.

**Figure 5:**
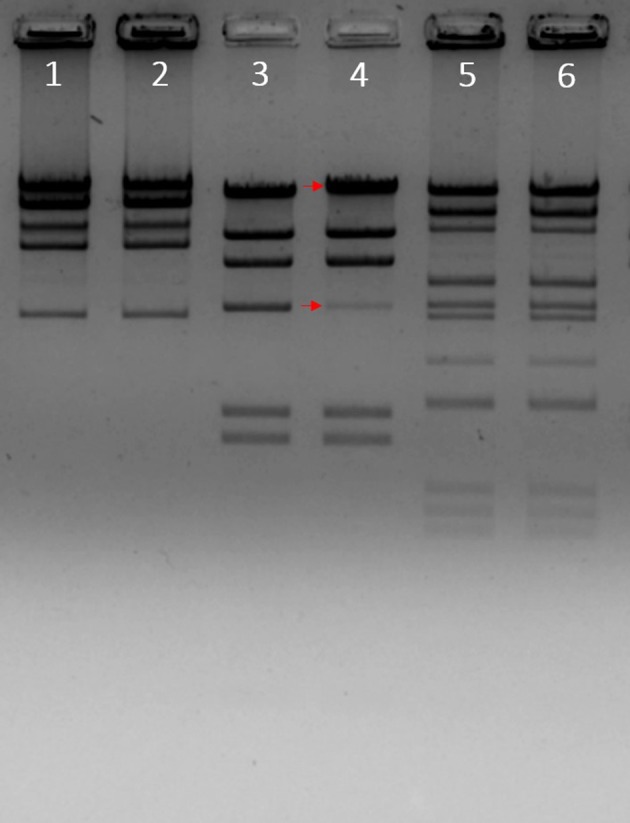
Enzymatic analysis of phiLLS genomic DNA. A restriction map of the genomic DNA of phage phiLLS was constructed using the restriction endonucleases *Bam*HI, *Hind*III, and the products were separated by agarose gel electrophoresis. Phage DNA digested with *Bam*HI without heat treatment (Lane 1), phage DNA digested with *Bam*HI with heat treatment (Lane 2). Genome of bacteriophage Lambda digested with *Hind*III was used as control, the size of the bands is indicated on the right and red arrows indicate the fragment containing the *cos* sequence (Lane 3). Phage DNA digested with *Hind*III without heat treatment (Lane 4), phage DNA digested with *Hind*III with heat treatment (Lane 5).

To determine whether phiLLS has cohesive ends, restriction enzyme digestion was performed and then analyzed by agarose gel electrophoresis (Figure 5). After the construction of the restriction map the estimated molecular size of phiLLS genome was of ~105 kb. The resulting restriction profile revealed no differences between banding profiles with and without prior annealing, suggesting the absence of cohesive ends in the DNA molecule of phiLLS (Casjens and Gilcrease, 2009). Moreover, analyses of several restriction profiles reveal the presence of submolar fragments. The appearance of submolar fragments in restriction enzyme digests indicates that phage phiLLS contain the *pac* site. This fragmentation is characteristic of phages that package their DNA by a headful mechanism; the initial packaging reaction starts at a terminase cleavage *pac* site sequence on a linear concatemer, and then successive packaging of phage concatemers into a procapsid by the terminase enzyme occurs (Auad et al., 1997).

Usually, virulent *pac*-type phages do not display generalized transduction due to a tendency by these phages to degrade enzymatically the genome of bacterial host. For example, phage T4 is a well-studied virulent *pac*-type phage that degrades the host DNA by the action of the nuclease and subsequently packages only phage DNA by the classical headful packaging mechanism (Streips and Yasbin, 2004; Bryan et al., 2016). Therefore, many *pac*-type phages have been described in recent years, and many of them have been proposed as biological control agents (Seal, 2013; Chang et al., 2015; Bardina et al., 2016).

### General features of the phage genome

To further our understanding of phage biology, the phage phiLLS genome was sequenced. A *de novo* genome assembly based on 12,903,357 paired-reads yielded a single contig with a high average coverage of >120X. The annotation of the properties of genome, such as positions, directions and putative functions of each gene are summarized in Table 2.

**Table 2:** Features of the open reading frames of bacteriophage phiLLS and homology to proteins databases.

**ORF**	**Start**	**Stop**	**Strand**	**Homology**	**Query cover (%)**	***E*-value**	**Identity (%)**
1	116	1,843	+	Putative tail tip protein (PHAGE_Entero_SSL2009a_NC_012223)	36	2.43E^−40^	90
2	1,899	2,141	−	Hypothetical protein [Shigella phage SHSML-45]	98	2.00E^−43^	85
3	2,119	2,565	−	Putative deoxyUTP pyrophosphatase [Escherichia phage T5]	91	2.00E^−98^	100
4	2,562	3,437	−	Flap endonuclease [Escherichia phage APCEc03]	99	0	99
5	3,437	3,919	−	D14 protein [Escherichia phage T5]	99	4.00E^−116^	100
6	3,923	5,761	−	Putative recombination endonuclease subunit D13 [Escherichia phage vB_EcoS_FFH1]	99	0	99
7	5,742	6,719	−	Calcineurin-like phosphoesterase superfamily domain protein [Escherichia phage slur09]	99	0	99
8	6,756	7,529	−	D11 protein [Escherichia phage vB_EcoS_FFH1]	99	0	100
9	7,522	7,806	−	Hypothetical protein T5.125 [Escherichia phage T5]	98	8.00E^−62^	100
10	8,027	9,379	−	Putative ATP-dependent helicase [Salmonella phage Spc35]	99	0	99
11	9,376	9,873	−	Hypothetical protein CPT_Shivani113 [Salmonella phage Shivani]	99	0	99
12	9,866	12,433	−	DNA polymerase [Escherichia phage vB_EcoS_FFH1]	99	0	99
13	12,527	12,856	−	Hypothetical protein SLUR09_00081 [Escherichia phage slur09]	99	9.00E^−62^	85
14	12,885	13,775	−	Putative DNA replication primase [Salmonella phage Spc35]	99	0	100
15	13,772	15,244	−	Putative replicative DNA helicase [Escherichia phage Akfv33]	99	0	100
16	15,327	16,094	−	Portal vertex protein [SHSML-45]	93	8.00E^−163^	100
17	16,087	16,866	−	NAD-dependent DNA ligase, subunit B [Escherichia phage vB_EcoS_FFH1]	100	0	98
18	17,069	18,040	−	NAD-dependent DNA ligase, subunit A [Escherichia phage T5]	99	0	99
19	18,041	18,313	−	Hypothetical protein			
20	18,400	18,708	−	Hypothetical protein T5.115 [Escherichia phage T5]	99	8.00E^−70^	100
21	18,759	19,055	−	Hypothetical protein T5.114 [Escherichia phage T5]	98	6.00E^−52^	100
22	19,092	19,502	−	D3 protein [Escherichia phage T5]	99	3.00E^−67^	100
23	19,606	19,857	−	Hypothetical protein [Escherichia phage vB_EcoS_FFH1]	98	2.00E^−51^	96
24	19,850	20,554	−	D2 protein [Escherichia phage T5]	99	8.00E^−171^	99
25	20,625	20,867	−	Hypothetical protein SPC35_0100 [Salmonella phage Spc35]	95	1.00E^−48^	99
26	20,842	23,631	−	Putative replication origin binding protein [Salmonella phage Spc35]	99	0	99
27	24,251	24,643	−	Hypothetical protein APCEc03_120 [Escherichia phage APCEc03]	99	5.00E^−88^	95
28	24,653	25,081	−	Hypothetical protein SLUR09_00096 [Escherichia phage slur09]	99	2.00E^−98^	98
29	25,084	25,590	−	Hypothetical protein [Escherichia phage vB_EcoS_FFH1]	99	2.00E^−109^	99
30	25,577	25,759	−	Hypothetical protein [Escherichia phage vB_EcoS_FFH1]	98	3.00E^−36^	98
31	25,750	26,574	−	Putative Sir2-like protein [Escherichia phage vB_EcoS_FFH1]	99	0	96
32	26,574	26,768	−	Hypothetical protein [Escherichia phage Akfv33]	98	6.00E^−36^	92
33	26,737	26,940	−	Hypothetical protein SPC35_0092 [Salmonella phage Spc35]	98	6.00E^−39^	100
34	26,937	27,296	−	Hypothetical protein APCEc03_129 [Escherichia phage APCEc03]	98	2.00E^−77^	97
35	27,395	29,269	−	Anaerobic ribonucleoside triphosphate reductase [Escherichia phage vB_EcoS_FFH1]	99	0	99
36	29,463	29,954	+	Putative HNH endonuclease family protein (PHAGE_Ralsto_RSK1_NC_022915)			
37	30,140	30,892	+	Phosphate starvation-inducible protein [Escherichia phage vB_EcoS_FFH1]	99	0	99
38	30,894	31,115	+	Tail length tape-measure protein 1 (PHAGE_Salmon_NR01_NC_031042)	98	2.00E^−45^	100
39	31,258	33,588	+	Aerobic ribonucleoside diphosphate reductase large subunit [Salmonella phage NR01]	97	0	99
40	33,690	34,190	+	Putative H-N-H-endonuclease P-TflVIII [Salmonella phage Spc35]	98	8.00E^−71^	66
41	34,190	35,335	+	Aerobic ribonucleoside diphosphate reductase, small subunit [Escherichia phage vB_EcoS_FFH1]	99	0	99
42	35,332	35,865	+	Putative dihydrofolate reductase [Escherichia phage APCEc03]	99	2.00E^−123^	97
43	35,865	36,704	+	Putative thymidylate synthase [Escherichia phage T5]	99	0	99
44	36,805	37,188	+	Hypothetical protein NR01_0022 [Salmonella phage NR01]	99	1.00E^−78^	95
45	37,181	37,729	+	Putative HNH endonuclease [Salmonella phage NR01]	97	2.00E^−71^	61
46	37,726	38,202	+	Ribonuclease H [Escherichia phage T5]	99	2.00E^−115^	99
47	38,279	38,557	+	Tail fibers protein	98	6.00E^−60^	100
48	38,641	39,156	+	Virion structural protein	80	5.00E^−98^	99
49	39,220	39,435	+	Baseplate wedge subunit	98	9.00E^−41^	99
50	39,567	39,713	+	Hypothetical protein NR01_0017 [Salmonella phage NR01]	97	8.00E^−27^	98
51	39,742	40,443	+	Putative metallopeptidase [Salmonella phage Spc35]	99	3.00E^−174^	99
52	40,514	40,696	+	Hypothetical protein SLUR09_00119 [Escherichia phage slur09]	98	2.00E^−34^	100
53	40,750	41,388	+	Hypothetical protein [Escherichia phage Akfv33]	99	4.00E^−149^	99
54	41,831	42,148	+	Hypothetical protein APCEc03_147 [Escherichia phage APCEc03]	99	9.00E^−72^	99
55	42,154	42,603	+	Cell wall hydrolase SleB [Escherichia phage Akfv33]	99	1.00E^−80^	100
56	42,672	42,842	+	Hypothetical protein SPC35_0072 [Salmonella phage Spc35]	98	2.00E^−30^	98
57	42,842	43,285	+	Hypothetical protein SPC35_0071 [Salmonella phage Spc35]	99	1.00E^−103^	100
58	44,245	45,192	+	Hypothetical protein [Escherichia phage vB_EcoS_FFH1]	99	1.00E^−179^	99
59	45,517	45,933	−	Hypothetical protein NR01_0007 [Salmonella phage NR01]	99	5.00E^−36^	98
60	45,955	46,938	−	Hypothetical protein [Escherichia phage vB_EcoS_FFH1]	99	2.00E^−177^	99
61	47,205	47,723	+	Hypothetical protein SPC35_0067 [Salmonella phage Spc35]	99	1.00E^−112^	100
62	47,937	48,125	+	Hypothetical protein [Escherichia phage vB_EcoS_FFH1]	98	2.00E^−36^	100
63	48,225	48,392	+	Hypothetical protein [Escherichia phage vB_EcoS_FFH1]	98	1.00E^−32^	100
64	48,385	48,591	+	Hypothetical protein [Escherichia phage vB_EcoS_FFH1]	98	5.00E^−40^	100
65	48,682	48,957	+	Hypothetical protein CPT_Shivani65 [Salmonella phage Shivani]	98	3.00E^−59^	98
66	49,505	49,777	+	Hypothetical protein SLUR09_00141 [Escherichia phage slur09]	98	5.00E^−48^	98
67	49,944	50,462	+	Hypothetical protein [Escherichia phage Bf23]	99	2.00E^−111^	98
68	50,619	50,804	+	Hypothetical protein T5.068 [Escherichia phage T5]	98	3.00E^−33^	98
69	50,916	51,263	+	Hypothetical protein NR01_0002 [Salmonella phage NR01]	99	2.00E^−77^	99
70	51,525	51,692	+	Hypothetical protein [Salmonella phage 5]	96	6.00E^−19^	70
71	51,899	52,057	+	Hypothetical protein [Salmonella phage 118970_sal2]	98	2.00E^−17^	69
72	52,244	52,411	+	Hypothetical protein [Salmonella phage 5]	98	4.00E^−31^	98
73	53,664	53,795	+	Hypothetical protein NR01_0148 [Salmonella phage NR01]	97	6.00E^−18^	81
74	53,810	54,004	+	Hypothetical protein [Escherichia phage Akfv33]	98	1.00E^−36^	91
75	54,133	54,753	+	No significant similarity found	−	–	−
76	54,834	55,085	+	Hypothetical protein APCEc03_006 [Escherichia phage APCEc03]	98	2.00E^−45^	100
77	55,078	55,242	+	Hypothetical protein APCEc03_007 [Escherichia phage APCEc03]	98	2.00E^−28^	96
78	55,402	55,692	+	Hypothetical protein [Escherichia phage vB_EcoS_FFH1]	98	1.00E^−62^	98
79	55,803	55,886	+	Hypothetical protein [Escherichia phage Bf23]	96	1.00E^−10^	100
80	55,994	56,188	+	Hypothetical protein [Escherichia phage Akfv33]	98	2.00E^−39^	100
81	56,231	56,599	+	Putative acetyltransferase-like protein [Salmonella phage NR01]	99	1.00E^−73^	98
82	56,681	56,995	+	Hypothetical protein [Escherichia phage vB_EcoS_FFH1]	99	1.00E^−60^	99
83	57,117	57,464	+	Hypothetical protein [Escherichia phage vB_EcoS_FFH1]	99	5.00E^−79^	100
84	57,541	57,822	+	Hypothetical protein [Escherichia phage vB_EcoS_FFH1]	98	6.00E^−61^	100
85	57,815	58,114	+	Hypothetical protein [Escherichia phage vB_EcoS_FFH1]	99	2.00E^−64^	98
86	58,107	58,502	+	Hypothetical protein [Escherichia phage vB_EcoS_FFH1]	99	3.00E^−82^	100
87	58,480	58,776	+	Hypothetical protein [Escherichia phage vB_EcoS_FFH1]	98	1.00E^−62^	94
88	58,773	59,057	+	Hypothetical protein [Escherichia phage vB_EcoS_FFH1]	98	3.00E^−61^	99
89	59,168	59,512	+	Hypothetical protein [Escherichia phage Akfv33]	99	3.00E^−63^	95
90	59,667	60,365	+	Hypothetical protein [Escherichia phage Akfv33]	99	2.00E^−171^	99
91	60,322	60,771	+	Putative terminase	99	4.00E^−90^	99
92	60,702	61,055	+	Hypothetical protein SLUR09_00180 [Escherichia phage slur09]	99	7.00E^−81^	97
93	61,055	61,807	+	Deoxynucleoside-5-monophosphate kinase [Escherichia phage APCEc03]	99	0	100
94	61,820	62,416	+	Putative ATP-dependent Clp protease [Escherichia phage vB_EcoS_FFH1]	99	4.00E^−145^	98
95	62,573	63,229	+	Holin [Salmonella phage Shivani]	99	8.00E^−159^	99
96	63,226	63,639	+	Lysozyme [Escherichia phage Akfv33]	99	8.00E^−97^	100
97	63,717	64,133	+	Hypothetical protein SLUR09_00005 [Escherichia phage slur09]	99	2.00E^−94^	100
98	64,209	64,640	+	Hypothetical protein T5.038 [Escherichia phage T5]	99	9.00E^−86^	100
99	64,633	64,923	+	Putative thioredoxin [Escherichia phage Akfv33]	98	4.00E^−65^	100
100	65,051	65,428	+	Hypothetical protein [Escherichia phage vB_EcoS_FFH1]	99	8.00E^−52^	100
101	65,433	65,678	+	Major capsid protein	98	6.00E^−48^	95
102	65,681	66,544	+	Serine/threonine-protein phosphatase 2 [Escherichia phage slur09]	99	0	99
103	66,544	66,843	+	Hypothetical protein [Escherichia phage vB_EcoS_FFH1]	99	1.00E^−66^	100
104	66,833	67,423	+	Putative serine/threonine protein phosphatase [Escherichia phage vB_EcoS_FFH1]	99	3.00E^−143^	100
105	67,416	67,538	+	No significant similarity found	−	–	–
106	67,591	68,022	+	Hypothetical protein T5.033 [Escherichia phage T5]	99	2.00E^−100^	99
107	68,101	68,352	+	Hypothetical protein T5.032 [Escherichia phage T5]	98	3.00E^−52^	100
108	68,352	68,513	+	Hypothetical protein SPC35_0029 [Salmonella phage Spc35]	98	5.00E^−26^	92
109	68,513	68,794	+	Tail sheath monomer	98	3.00E^−54^	95
110	68,791	69,036	+	Hypothetical protein SPC35_0027 [Salmonella phage Spc35]	98	2.00E^−49^	98
111	69,026	69,352	+	Hypothetical protein APCEc03_041 [Escherichia phage APCEc03]	99	1.00E^−70^	97
112	69,452	69,652	+	Hypothetical protein SPC35_0025 [Salmonella phage Spc35]	98	2.00E^−38^	98
113	69,649	70,110	+	Hypothetical protein SLUR09_00019 [Escherichia phage slur09]	99	1.00E^−109^	99
114	70,058	70,429	+	Hypothetical protein T5.025 [Escherichia phage T5]	99	1.00E^−80^	98
115	70,410	70,688	+	Hypothetical protein [Escherichia phage vB_EcoS_FFH1]	72	6.00E^−42^	100
116	70,690	71,136	+	Putative terminase	99	1.00E^−108^	99
117	71,129	71,419	+	Hypothetical protein [Escherichia phage vB_EcoS_FFH1]	98	6.00E^−63^	98
118	71,407	71,640	+	Minor tail protein	98	1.00E^−45^	99
119	71,640	71,825	+	Hypothetical protein [Escherichia phage vB_EcoS_FFH1]	45	3.00E^−09^	96
120	71,825	72,385	+	Hypothetical protein APCEc03_050 [Escherichia phage APCEc03]	99	2.00E^−132^	94
121	72,522	73,406	+	Hypothetical protein T5.018 [Escherichia phage T5]	99	0	99
122	75,140	75,343	−	Hypothetical protein [Escherichia phage vB_EcoS_FFH1]	98	1.00E^−41^	100
123	75,362	75,520	−	Hypothetical protein			
124	75,517	75,861	−	Hypothetical protein [Escherichia phage vB_EcoS_FFH1]	99	2.00E^−78^	99
125	75,863	76,075	−	Hypothetical protein T5.014 [Escherichia phage T5]	98	1.00E^−42^	100
126	76,078	76,227	−	Hypothetical protein [Escherichia phage vB_EcoS_FFH1]	98	5%	92
127	76,274	76,504	−	Hypothetical protein [Escherichia phage Akfv33]	98	7.00E^−49^	97
128	76,622	77,626	−	DNA N-6-adenine methyltransferase	96	4.00E^−176^	87
129	78,765	78,968	+	Hypothetical protein NR01_0098 [Salmonella phage NR01]	98	2.00E^−40^	100
130	79,197	79,448	+	Baseplate wedge protein	98	2.00E^−41^	96
131	79,547	79,954	+	Virion structural protein	99	6.00E^−92^	99
132	80,013	80,210	+	Putative membrane protein [Enterobacteria phage DT57C]	98	4.00E^−36^	94
133	80,307	81,977	+	Baseplate wedge subunit	99	0	97
134	82,051	82,443	+	Baseplate wedge subunit	99	4.00E^−82^	90
135	82,518	83,237	+	Minor tail protein	98	4.00E^−174^	97
136	83,402	83,647	−	Hypothetical protein SPC35_0145 [Salmonella phage Spc35]	98	5.00E^−40^	96
137	83,640	83,831	−	Tail assembly protein	98	2.00E^−15^	88
138	83,828	83,944	−	Tail fiber protein	97	2.00E^−06^	97
139	83,935	84,063	−	Hypothetical protein [Escherichia phage Akfv33]	97	2.00E^−10^	100
140	84,240	84,506	−	Receptor-blocking protein [Escherichia phage Akfv33]	98	2.00E^−58^	100
141	84,592	86,349	+	Super-infection exclusion protein	99	0	98
142	86,360	86,842	+	Hypothetical protein SLUR09_00049 [Escherichia phage slur09]	99	1.00E^−79^	99
143	86,842	88,158	+	Terminase, large subunit [Escherichia phage T5]	99	0	99
144	88,273	88,710	+	Hypothetical protein APCEc03_075 [Escherichia phage APCEc03]	99	2.00E^−91^	100
145	88,710	89,927	+	Portal protein [Escherichia phage vB_EcoS_FFH1]	99	0	99
146	89,924	90,418	+	Tail fibers protein [Shigella phage SHSML-45]	99	1.00E^−93^	99
147	90,422	91,054	+	Putative prohead protease [Escherichia phage vB_EcoS_FFH1]	99	9.00E^−155^	100
148	91,072	92,448	+	Major head protein precursor [Escherichia phage T5]	99	0	98
149	92,508	93,020	+	Hypothetical protein APCEc03_080 [Escherichia phage APCEc03]	99	4.00E^−124^	100
150	93,020	93,787	+	Hypothetical protein CPT_Shivani137 [Salmonella phage Shivani]	99	0	99
151	93,791	94,276	+	Hypothetical protein [Escherichia phage vB_EcoS_FFH1]	99	5.00E^−117^	100
152	94,303	95,709	+	Putative major tail protein [Escherichia phage vB_EcoS_FFH1]	99	0	99
153	95,714	96,616	+	Minor tail protein [Escherichia phage vB_EcoS_FFH1]	99	0	99
154	96,609	97,013	+	Hypothetical protein [Escherichia phage vB_EcoS_FFH1]	99	5.00E^−94^	99
155	97,111	97,443	+	Hypothetical protein [Escherichia phage Akfv33]	99	2.00E^−63^	100
156	97,527	101,207	+	Pore-forming tail tip protein [Escherichia phage Akfv33]	99	0	99
157	101,317	101,931	+	DNA polymerase I	99	1.00E^−144^	99
158	101,928	104,777	+	Tail protein Pb3 [Escherichia phage vB_EcoS_FFH1]	99	0	99
159	104,777	106,834	+	Tail protein [Escherichia phage APCEc03]	99	0	98
160	106,840	107,262	+	Putative phage tail protein [Escherichia phage Akfv33]	99	4.00E^−95^	97

**Figure 6:**
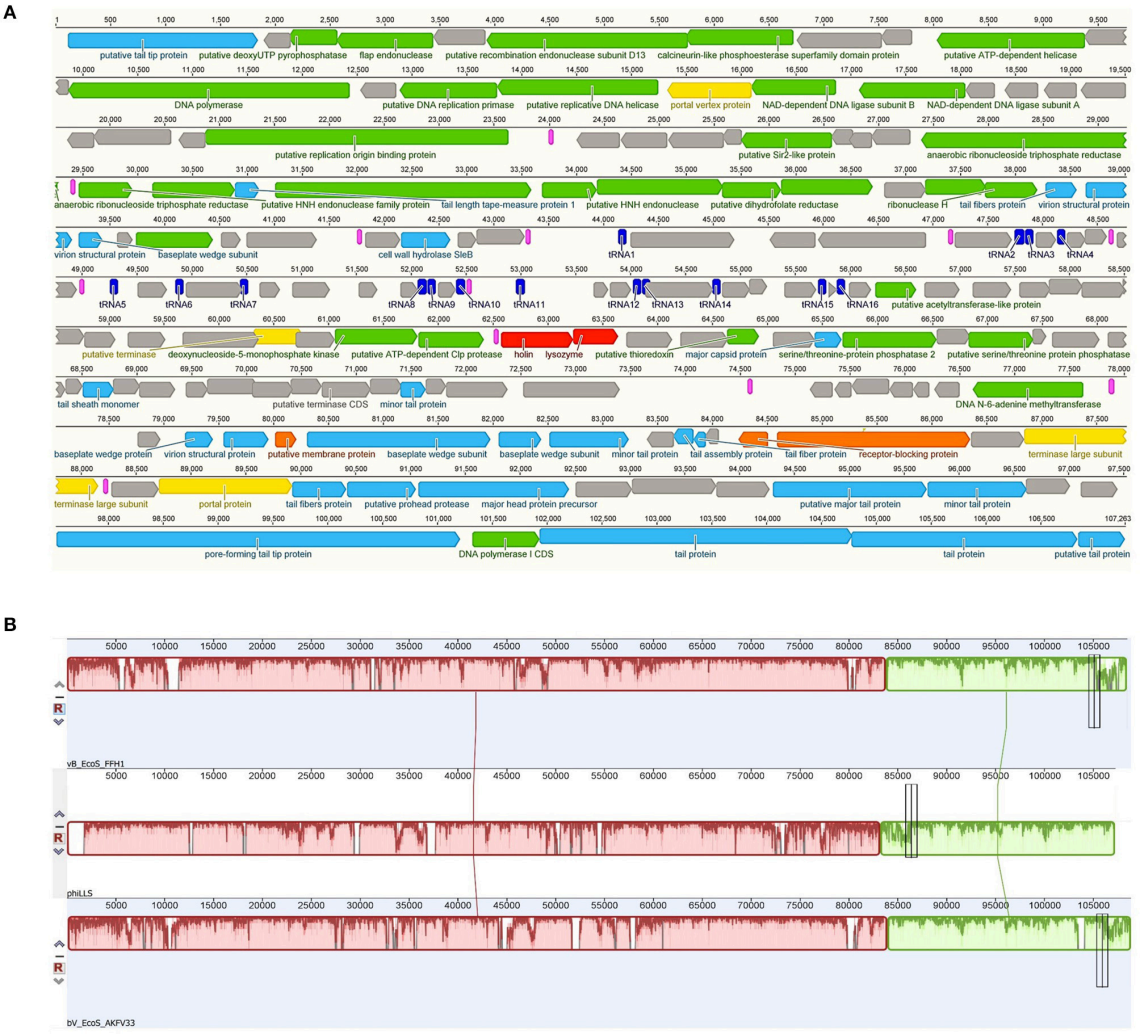
Map of the genome organization of bacteriophage phiLLS **(A)** and Comparative genomic maps of phages phiLLS, vB_EcoS_FFH1 and bV_EcoS_AKFV33 using the Mauve progressive alignments to determine conserved sequence regions **(B)**. **(A)** The predicted ORFs are indicated as arrows, the orientation of which shows the direction of transcription. Different colors identify predicted molecular function for ORF. DNA regulation module (Green arrows), packaging module (yellow arrows), phage structural proteins (blue arrows), host lysis proteins (red arrows), hypothetical proteins (black arrows), and accessory genes (orange arrows). Other genetic elements are shown, including putative promoters (pink), and tRNAs (dark blue). **(B)** Boxes with identical colors represent local colinear blocks (LCB), indicating homologous genomic regions shared by phage chromosomes without sequence rearrangements.

The genome of phiLLS is double-stranded DNA genome consisting of 107,263 bp with a GC content of 39.0%. In total, 160 putative ORFs were predicted in phage genome, with 112 ORFs on the positive strand and 48 ORFs on the negative strand (Figure 6A). The average gene length is 579 bp, with sizes ranging from 84 to 3,681 nucleotides. A total of 93,908 nucleotides (87.6% of the genome) were involved in coding for putative proteins. Only 69 ORFs (31.34%) were predicted and determined to be functional, whereas 91 were assigned to hypothetical proteins based on the assumption that sequence homology reflects a functional relationship.

Based on the result of BLAST analyses, the predicted amino acid sequences from 42 ORFs of phiLLS display significant similarity to the T5-like phages, especially to the coliphages vB_EcoS_FFH1 (GenBank accession number: NC_024139.1) (share a nucleotide identity of 96%) and bV_EcoS_AKFV33 (HQ665011.1) (94 %). The three phages were isolated in different regions around the world, suggesting the complex evolutionary relationships among these phages (Shen et al., 2016). Moreover, Mauve alignment of the threes phages showed that some regions are highly homologous, with no significant rearrangements observed, suggesting high level of nucleotide identity and lack of major rearrangements (Figure 6B). This indicated that these phages have a common genome organization and gene arrangement.

Furthermore, the BLAST analysis indicates that the phages phiLLS, vB_EcoS_FFH1, and bV_EcoS_AKFV33, are related phylogenetically with a minimum 87% of query cover and, 70% shared orthologous proteins. The genetic similarities among these phages may correlate with their biological properties because the conserved core genes include the replication and morphogenesis modules of each genome, interestingly these bacteriophages are effective in limiting contamination with *E. coli* (Hong et al., 2014), suggesting that phage phiLLS may show promise as a biological control agent. The conservation of genes among the four phage genomes may indicate that the phages retained ancestral structural genes to maintain their infective capacity to stablish infective cycle on bacterial hosts (Merrill et al., 2014). In contrast, the ls_1 tail protein encoded by phiLLS show a greater divergence. The tail proteins are thought to be involved in host recognition, and confer the phage host range specificity. In regard, these three phages share high DNA sequence homology but could exhibit different host specificities, which the small different in tail fiber proteins are often associated with significant differences in host ranges and other biological properties.

**Figure 7:**
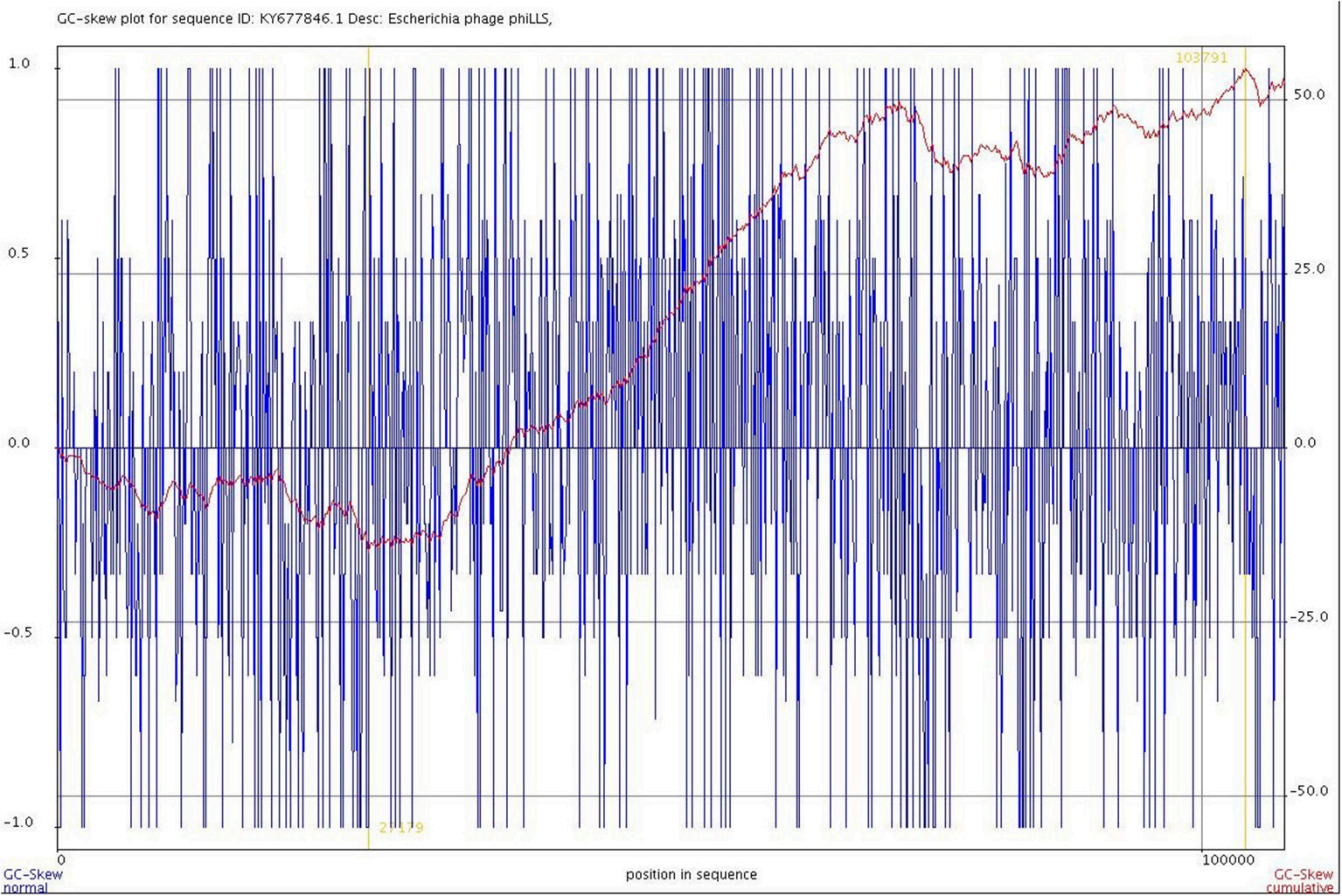
Cumulative GC skew analysis of the phage genome sequence. The global minimum and maximum are displayed in the cumulative graph were calculated by using a window size of 1,000 bp and a step size of 100 bp. The GC-skew and the cumulative GC-skew are represented by blue and red lines, respectively. The minimum and maximum of a GC-skew can be used to predict the origin of replication (27179 nt) and the terminus location (103791 nt).

In an attempt to define the origin and terminus of replication of the phage genome, a cumulative GC skew analysis was performed. The results of GC skew analysis in the genome of the phiLLS phage (Figure 7) indicate that the origin of replication is in the position 27,179 nt, and a replication terminus could be located in the region 103,791 nt because two inflection points were identified, indicating an asymmetric base composition, which are the lowest in origin and highest in terminus (Uchiyama et al., 2008). Our analysis showed that the origin of replication is flanked by direct repeat structures and is adjacent to a gene encoding a replication protein, which give greater support to our results.

Based on the information obtained from an exhaustive search of the NCBI GenBank database, it was possible to determine that the coliphages have very different genome sizes (Supplementary Material 1), which agrees with reports for most bacteriophages (Hatfull, 2008). The available data show that coliphages have a range of genome sizes from 3.393 kb (phage BZ13; GenBank accession number FJ483838) to 348.532 kb (phage 121Q; GenBank accession number KM507819). However, only a small number of the reported *Siphoviridae* coliphages have genomes sizes larger than 100 kb, such as DT57C, DT571/2, vB_EcoS_FFH1, Akfv33, Eps7, and T5 deposited in the GenBank under accession numbers KM979354, KM979355, KJ190157, HQ665011, CP000917, and AY543070, respectively.

The phages within the family *Siphoviridae* have average genome sizes of 53.70 kb. It is therefore surprising that phiLLS has an unusually large genome size for a member of this family of bacteriophages. Typically, larger phage genomes contain more genes, reflecting the more complex virion structure, and encode considerable number of enzymes associated with viral replication during the infection cycle (Brown, 2012).

The bacteriophage phiLLS genome has a high gene density—1.64 genes per kilobase. The genome analysis suggests that the phage phiLLS is strictly lytic and does not carry genes associated with virulence factors and/or potential immunoreactive allergens in their genomes. Therefore, this phage has desirable genetic features as a biocontrol agent. However, further oral toxicity testing is needed to ensure the safety of phage use.

The molecular GC content was calculated at 39.0%, which is significantly lower than of *E. coli* (average 50%). The lower GC content of phage phiLLS may suggest an adaptive strategy to optimize gene expression of the viral genome. This feature is favorable to activate gene transcription, perhaps because the GC content is generally lower in virulent phages than in their hosts (Rocha and Danchin, 2002; Zuber et al., 2007; Lucks et al., 2008).

The phiLLS genome is organized in a modular gene structure that is common of tailed bacteriophage genomes (Krupovic et al., 2011) and each module includes groups of genes involved in the same biological pathways or in related biological functions, consisting of structure/morphogenesis, DNA packaging, cell lysis, DNA metabolism and replication modules.

**Figure 8:**
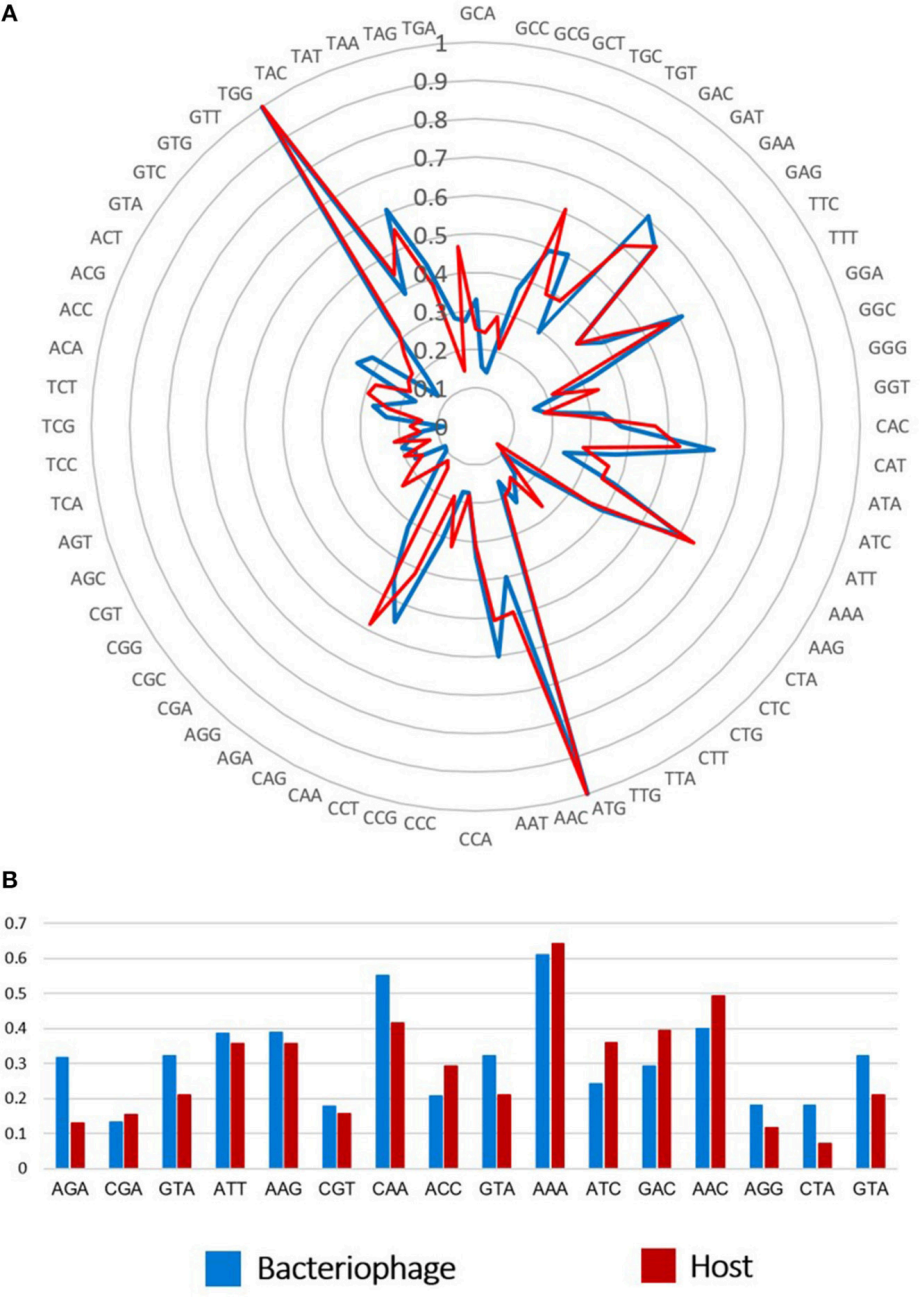
Comparison of codon usage and tRNAs between phiLLS and host. **(A)** Rose plot show the possible association between tRNAs and codon usage in phage and their host. The frequency scale is represented at the center of the rose plot. **(B)** Ten tRNAs present in phage genome tend to correspond to codons that are highly used by the phage genes, while rare in the host genome.

In addition, the phiLLS genome was found to contain 16 tRNA genes with anticodons for Arg, Ser, Met, Leu, Glu, Cys, Asn, Pro, Lys, Gln, Gly, and Ile, located around a region at position 44,136–55,956 bp of the genome (Figure 8). Ten phage-encoded tRNAs corresponded to codons that are more abundant in the phage than in the host. Presumably, the tRNAs encoded in the phiLLS genome would counter a deficiency of codon usage in the host during translation. The presence of tRNA genes in genomes of bacteriophages may possibly be associated with a significant difference in codon usage and GC content between phages and their hosts (Limor-Waisberg et al., 2011). Moreover, the codon usage of phage phiLLS exceeded that of the host on 10 predicted tRNAs present in the phage. This suggests that phiLLS could supply specific tRNAs on its own in the event of a tRNA deficiency, potentially indicating a strategy for translational efficiency (Uchiyama et al., 2008; Bahir et al., 2009). According to Bailly-Bechet et al. (2007), lytic phages especially encode many tRNA genes to ensure optimal translation and therefore may replicate faster.

In summary, phage phiLLS genome sequence analysis revealed valuable information concerning its biology. Detailed genomic analysis showed a modular organization, which is different from other identified enterobacteriophages. Nevertheless, it demonstrated a high degree of identity with ORFs from some other phages, especially with T5-like bacteriophages. Moreover, the phiLLS phage does not encode lysogenic genes. The phiLLS genome encodes several putative proteins with lytic activity, which may be exploited for other biotechnological applications. This study identified the groups of enzymes responsible for producing bacterial lysis. The practical use of the phiLLS genome will be derived from the understanding of its organization. Based on the genetic information of this phage, future work may be performed to obtain enzymes with antimicrobial activity for the biocontrol of pathogenic bacteria.

In conclusion, we have isolated and characterized a new lytic phage, phiLLS, with lytic activity against multidrug-resistant *E. coli* isolates. The newly isolated phage is characterized by a broad host range and belongs to family *Siphoviridae*. Furthermore, this phage exhibited a large burst size of 176 plaque-forming units per infected cell. Moreover, the genome sequence analysis of phiLLS provided no evidence of lysogenic genes (obligately lytic), genes related to potential virulence factors, antibiotic resistance genes, toxins or potential immunoreactive food allergens. Based on all these characteristics, phage phiLLS is a suitable and promising candidate as a biocontrol agent. However, further oral toxicity testing and *in vivo* trials are needed to ensure the safety of phage use.

## Author note

6 Preliminary challenge trials were performed to evaluate the potential of the isolated phages as 7 antimicrobials against *S. epidermidis*. The mixture of the temperate phages phi-IPLA6 and 8 phi-IPLA7 was used at MOI = 10 to infect S. epidermidis F12 (Figure 4A). Within the first 4 h, 9 viable counts were similar to phage-infected and uninfected (control) cultures, and 10 staphylococcal proliferation was prevented afterwards. S. epidermidis counts were reduced by 11 2.27 log units compared with the control cultures (Figure 4A). Furthermore, viable bacteria were still at 106 CFU ml-1 12 at 24 h (data not shown). It was expected that the addition of a mixture of 13 the two temperate phages to *S. epidermidis* cultures would suppress bacterial growth and even 14 fully lyse the host culture since they do not belong to the same immunitygroup.

## Author contributions

LA, LR, and JL conceived, designed and coordinated the study. LA, LR, and AG carried out the experimentation. LA, LR, and JL analyzed the results. Contributed reagents/materials/analysis tools: JL, CC, and AG. CC edited the English grammar of the manuscript. All authors wrote, read and approved the final manuscript.

### Conflict of interest statement

The authors declare that the research was conducted in the absence of any commercial or financial relationships that could be construed as a potential conflict of interest.
